# Cardio-immuno-oncology in cardiac tumors and cardiac metastases: immune microenvironment, therapeutic opportunities, and cardiotoxicity

**DOI:** 10.3389/fimmu.2026.1877480

**Published:** 2026-09-18

**Authors:** Hao He, Jianyu Xiong, Junxiang Wang, Jianglin Wang, Jiayao Li, Zhongxiao Lin

**Affiliations:** 1 Department of Cardiology, Shenzhen Longgang Second People’s Hospital, Shenzhen, Guangdong, China; 2 Guangzhou Municipal and Guangdong Provincial Key Laboratory of Molecular Target and Clinical Pharmacology, School of Pharmaceutical Sciences, Guangzhou Medical University, Guangzhou, China; 3 Key Laboratory of Biological Targeting Diagnosis, Therapy and Rehabilitation of Guangdong Higher Education Institutes, The Fifth Affiliated Hospital, Guangzhou Medical University, Guangdong, Guangzhou, China

**Keywords:** cardiac metastasis, cardiac tumors, cardio-oncology, immune checkpoint inhibitors, tumor immune microenvironment

## Abstract

Primary cardiac tumors are rare, whereas cardiac metastases are more common forms of cardiac involvement in patients with advanced malignancies; both are clinically important because of their potential cardiovascular consequences. This review summarizes current evidence on the immune microenvironment, immune-evasion mechanisms, immunotherapeutic opportunities, multi-omics-based immune decoding, and cardiovascular toxicity in cardiac tumors and cardiac metastases. Cardiac myxoma is discussed as a benign primary cardiac tumor with inflammatory, cytokine-mediated, metabolic, and candidate immune-regulatory features, whereas malignant primary cardiac tumors, particularly cardiac sarcomas such as cardiac angiosarcoma, are reviewed in relation to immunosuppressive niches, macrophage-rich microenvironments, T-cell dysfunction, and heterogeneous responses to immune checkpoint inhibitors. Cardiac metastases are considered as a clinically relevant setting in which immunotherapy may be feasible for selected patients with careful cardiovascular assessment. Key mechanisms discussed include immune checkpoint pathways, tumor-associated macrophages, metabolic reprogramming, cytokine signaling, vascular abnormalities, and spatial immune organization. The review also highlights the role of single-cell sequencing, spatial transcriptomics, proteomics, metabolomics, and integrative multi-omics approaches in defining tumor-specific immune landscapes and potential biomarkers. Finally, immune checkpoint inhibitor-associated cardiotoxicity, particularly myocarditis, is discussed with emphasis on baseline cardiovascular assessment, biomarker monitoring, multimodality imaging, and multidisciplinary cardio-oncology management.

## 1 Introduction

Primary malignant cardiac tumors are exceptionally rare, whereas secondary cardiac involvement by metastatic malignancies is considerably more common. However, tumors involving the heart, whether primary cardiac tumors or cardiac metastases, can cause disproportionate clinical harm because even small lesions may disrupt intracardiac blood flow, cardiac rhythm, myocardial function, or pericardial homeostasis. Therefore, primary cardiac tumors and cardiac metastases represent clinically important forms of cardiac neoplastic involvement at the intersection of oncology, cardiovascular medicine, imaging, pathology, and immunology. Malignant primary cardiac tumors and cardiac metastases are associated with aggressive clinical behavior and often require complex multidisciplinary management (1, 2).

Traditionally, the study of cardiac tumors has focused on clinical presentation, imaging diagnosis, surgical treatment, pathological classification, and prognosis. Cardiac myxoma, as the most common benign primary cardiac tumor, has been mainly discussed in relation to obstruction, embolization, inflammatory manifestations, and surgical resection. In contrast, primary cardiac sarcomas, particularly cardiac angiosarcoma, are characterized by invasive growth, early metastasis, limited therapeutic options, and poor outcomes (1, 3–6). However, these conventional perspectives do not fully explain the biological heterogeneity of cardiac tumors, their variable clinical behavior, or their differential responses to systemic therapy.

In recent years, the tumor microenvironment (TME) has emerged as a key determinant of tumor progression and treatment response. The immune contexture of tumors, including the density, composition, localization, and functional state of immune infiltrates, can strongly influence clinical outcomes and therapeutic sensitivity (7, 8). Immune checkpoint pathways, tumor-associated macrophages, cytokine networks, metabolic reprogramming, vascular abnormalities, and spatial immune organization collectively shape tumor immune behavior and may contribute to immune escape. These concepts have been extensively investigated in common solid tumors, but their relevance to cardiac tumors remains insufficiently explored. This knowledge gap has direct clinical relevance because patients with advanced malignancies and cardiac involvement may increasingly receive immune-based therapies, while tumor-related cardiac abnormalities may complicate treatment selection, cardiovascular surveillance, and recognition of treatment-related toxicity.

Recent advances in single-cell sequencing, spatial transcriptomics, proteomics, metabolomics, and integrative multi-omics analysis have provided new opportunities to decode tumor immunity at higher resolution. Unlike conventional histopathology or bulk molecular profiling, these approaches can identify rare immune cell subsets, spatially organized immune niches, tumor–stromal interactions, metabolic immune suppression, and clinically relevant immune-evasion pathways. For cardiac tumors, which are constrained by rarity, limited tissue availability, small sample sizes, and anatomical complexity, multi-omics approaches may be particularly valuable for defining disease-associated immune landscapes, identifying candidate biomarkers, and generating hypotheses for subsequent biological and clinical validation (8–12).

Emerging studies suggest that cardiac tumors possess distinct and biologically meaningful immune microenvironments. In cardiac myxoma, inflammatory cell infiltration, macrophage polarization, cytokine signaling, autophagy-related inflammatory regulation, and single-cell evidence of immune-evasion mechanisms such as the SIRPα–CD47 axis indicate that this traditionally “benign” tumor may have a more active immune and stromal biology than previously appreciated (12–15). In malignant cardiac tumors, particularly primary cardiac sarcomas such as cardiac angiosarcoma, recent single-cell and clinical studies have begun to reveal suppressive immune niches, macrophage-rich microenvironments, exhausted T-cell states, and limited but heterogeneous responses to immune checkpoint inhibitors (9, 16, 17). These findings suggest that immune regulation may be relevant not only to tumor progression but also to therapeutic vulnerability. However, the current evidence base remains limited by small cohorts, tumor-type heterogeneity, and the rarity of direct mechanistic studies in cardiac tumors. Accordingly, findings derived directly from cardiac tumors should be distinguished from mechanistic concepts extrapolated from more common extracardiac malignancies.

The increasing clinical use of immunotherapy further highlights the need to understand cardiac tumors from a cardio-immuno-oncology perspective. In this review, cardio-immuno-oncology refers to the intersection of tumor immunity, immunomodulatory cancer therapy, and cardiovascular biology. Unlike conventional cardio-oncology, which primarily addresses the cardiovascular consequences of cancer and anticancer treatment, this framework emphasizes how immune mechanisms may simultaneously influence tumor behavior, therapeutic response, and cardiovascular injury. Immune checkpoint inhibitors have transformed the treatment of many malignancies, and selected patients with primary cardiac sarcomas or cardiac metastases may derive clinical benefit (17–19). Nevertheless, responses remain modest and heterogeneous, and immunotherapy carries the risk of immune-related cardiovascular toxicities, including myocarditis, pericardial disease, vasculitis, arrhythmias, and non-inflammatory myocardial dysfunction (20–23). In patients with cardiac tumors or cardiac metastases, these toxicities are particularly challenging because tumor-related cardiac involvement may overlap clinically with treatment-related cardiotoxicity.

Therefore, a comprehensive review of the immune microenvironment and immunotherapy of cardiac tumors is needed. This article summarizes the current understanding of primary cardiac tumors and cardiac metastases, with emphasis on immune landscape, immune-evasion mechanisms, immunotherapeutic evidence, cardiovascular toxicity, and future directions. Rather than considering primary cardiac tumors, cardiac metastases, tumor immunity, and treatment-related cardiovascular toxicity as separate topics, we integrate these dimensions within a unified cardio-immuno-oncology framework. We also distinguish direct cardiac tumor-specific evidence from observations extrapolated from extracardiac malignancies and identify areas in which current evidence remains exploratory or hypothesis-generating. By integrating tumor immunology with cardio-oncology, this review aims to provide a framework for understanding the immune-regulated features of cardiac tumors and to identify priorities for future mechanistic and translational research in precision cardio-immuno-oncology.

## 2 Classification and clinical impact of cardiac tumors and cardiac metastases

Cardiac tumors are uncommon lesions with diverse histological origins, clinical manifestations, and biological behaviors. They can be broadly classified into primary cardiac tumors, which arise from cardiac tissues, and secondary cardiac tumors, also referred to as cardiac metastases, which result from metastatic spread from extracardiac malignancies. Although primary cardiac tumors are rare, they are clinically important because even histologically benign tumors may cause serious complications due to their intracardiac location, including obstruction, embolization, arrhythmias, and heart failure-like symptoms (1, 3).

### 2.1 Primary cardiac tumors

Primary cardiac tumors are rare, and most are benign. Among benign primary cardiac tumors, cardiac myxoma is the most common subtype in adults. It usually occurs in the atria, particularly the left atrium, and may present with obstructive symptoms, embolic events, or constitutional manifestations such as fever, weight loss, and elevated inflammatory markers. Surgical resection remains the standard treatment for symptomatic or high-risk cardiac myxoma, and prognosis is generally favorable after complete removal (3, 4, 24).

Malignant primary cardiac tumors are much less common than benign tumors, but they are associated with aggressive behavior and poor prognosis. Primary cardiac sarcomas account for the majority of malignant primary cardiac tumors, with angiosarcoma, undifferentiated sarcoma, leiomyosarcoma, and intimal sarcoma among the major histological subtypes. Cardiac angiosarcoma often involves the right atrium and is characterized by early local invasion, metastatic potential, and limited survival. In contrast, other sarcoma subtypes may occur more frequently in the left atrium or great vessels, leading to different clinical presentations and treatment challenges (1, 5, 6).

Diagnosis of primary cardiac tumors requires integration of clinical assessment, imaging, pathology, and, increasingly, molecular characterization. Echocardiography is commonly used as an initial diagnostic tool, whereas cardiac magnetic resonance imaging (CMR) and computed tomography provide additional information regarding tumor location, tissue characteristics, infiltration, and extracardiac extension. Histopathological examination remains essential for definitive diagnosis and classification, particularly when distinguishing benign tumors from malignant tumors or thrombus-like lesions (1, 3, 6).

### 2.2 Cardiac metastases

Cardiac metastases are more common than primary malignant cardiac tumors and usually occur in the setting of advanced systemic malignancy. They may arise from various extracardiac cancers, including lung cancer, breast cancer, melanoma, lymphoma, renal cell carcinoma, and other solid tumors. Rare cardiac metastases have also been reported from tumors such as Merkel cell carcinoma and nasopharyngeal carcinoma, particularly in the context of immunotherapy-sensitive disease (19, 25). Metastatic involvement can affect the pericardium, myocardium, endocardium, or intracavitary structures, with the pericardium being a frequent site of involvement (1, 2).

The clinical manifestations of cardiac metastases are highly variable. Some patients remain asymptomatic, and cardiac involvement may be detected incidentally by imaging or at autopsy. Others may develop pericardial effusion, cardiac tamponade, arrhythmias, conduction abnormalities, embolic complications, intracardiac obstruction, or heart failure-like symptoms. Because these manifestations often overlap with other cardiovascular diseases or treatment-related cardiotoxicity, diagnosis can be challenging and usually requires multimodality imaging (2, 22).

The prognosis of cardiac metastasis depends on the primary tumor type, systemic disease burden, location and extent of cardiac involvement, and feasibility of continued anticancer therapy. Retrospective data suggest that continued antitumor treatment after the diagnosis of cardiac metastasis may improve survival in selected patients, indicating that cardiac metastasis should not always be viewed as an absolute endpoint of oncological treatment (2). With the increasing use of immunotherapy and targeted therapy, understanding the biological and clinical features of cardiac metastases has become increasingly important for individualized treatment planning and cardio-oncology management (18, 19).

### 2.3 Clinical impact on the heart

Although primary cardiac tumors are rare and cardiac metastases are comparatively more common, both can have a clinical impact disproportionate to tumor size because of the functional importance of cardiac structures. Tumor involvement of the heart may lead to intracardiac obstruction, systemic or pulmonary embolism, arrhythmias, conduction abnormalities, pericardial effusion, cardiac tamponade, myocardial infiltration, and heart failure-like symptoms. In addition, cardiac tumor-related manifestations may overlap with treatment-related cardiotoxicity, particularly in patients receiving immunotherapy, radiotherapy, targeted therapy, or other cardiotoxic anticancer treatments. Therefore, classification of cardiac tumors should be considered together with their potential cardiovascular consequences (1–3, 22).

The major categories of cardiac tumors and cardiac metastases, together with their main clinical impacts on the heart, are summarized in **Figure 1**.

**Figure 1:**
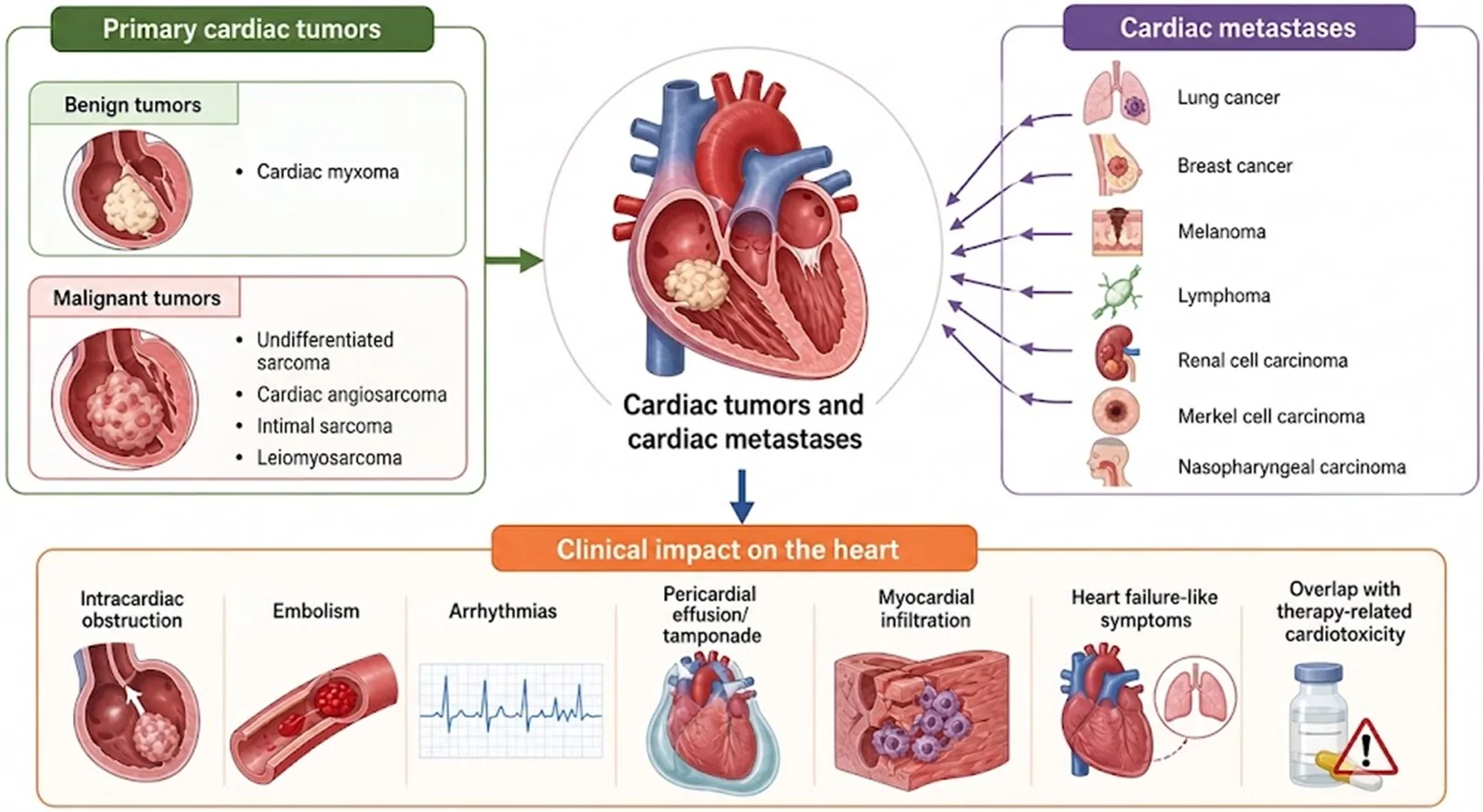
Classification and clinical impact of cardiac tumors and cardiac metastases. Cardiac tumors can be broadly divided into primary cardiac tumors and cardiac metastases. Primary cardiac tumors include benign lesions, such as cardiac myxoma, and malignant tumors, most commonly cardiac sarcomas, with major subtypes including angiosarcoma, undifferentiated sarcoma, intimal sarcoma, and leiomyosarcoma. Cardiac metastases arise from extracardiac malignancies, including lung cancer, breast cancer, melanoma, lymphoma, renal cell carcinoma, Merkel cell carcinoma, and nasopharyngeal carcinoma. Although primary cardiac tumors are rare, cardiac metastases are more common; both may cause clinically significant cardiovascular complications, including intracardiac obstruction, embolism, arrhythmias, pericardial effusion or tamponade, myocardial infiltration, heart failure-like symptoms, and diagnostic overlap with therapy-related cardiotoxicity.

## 3 Immune landscape and immune evasion in cardiac tumors

Cardiac tumors develop within a complex and dynamic tumor microenvironment (TME), which comprises tumor cells, stromal components, and a diverse array of immune cell populations. Increasing evidence suggests that the composition, density, and spatial organization of immune infiltrates critically influence tumor progression and clinical outcomes. The concept of “immune contexture” emphasizes that not only the presence but also the localization and functional orientation of immune cells determine their impact on tumor behavior (7).

Recent advances in tumor immunology have further demonstrated that the TME is highly heterogeneous across tumor types and patients, and that distinct immune phenotypes are associated with differential responses to immunotherapy (8). However, compared with extensively studied solid tumors, the immune microenvironment of cardiac tumors remains relatively underexplored, and current understanding is largely extrapolated from other tumor systems. In this context, emerging evidence indicates that benign and malignant cardiac tumors exhibit fundamentally different immune landscapes, ranging from inflammation-dominated environments to profoundly immunosuppressive niches. Elucidating these differences is essential for understanding disease mechanisms and identifying potential therapeutic strategies. The integrated cardio-immuno-oncology framework linking immune suppression, therapeutic intervention, and cardiovascular risk is summarized in **Figure 2**.

**Figure 2:**
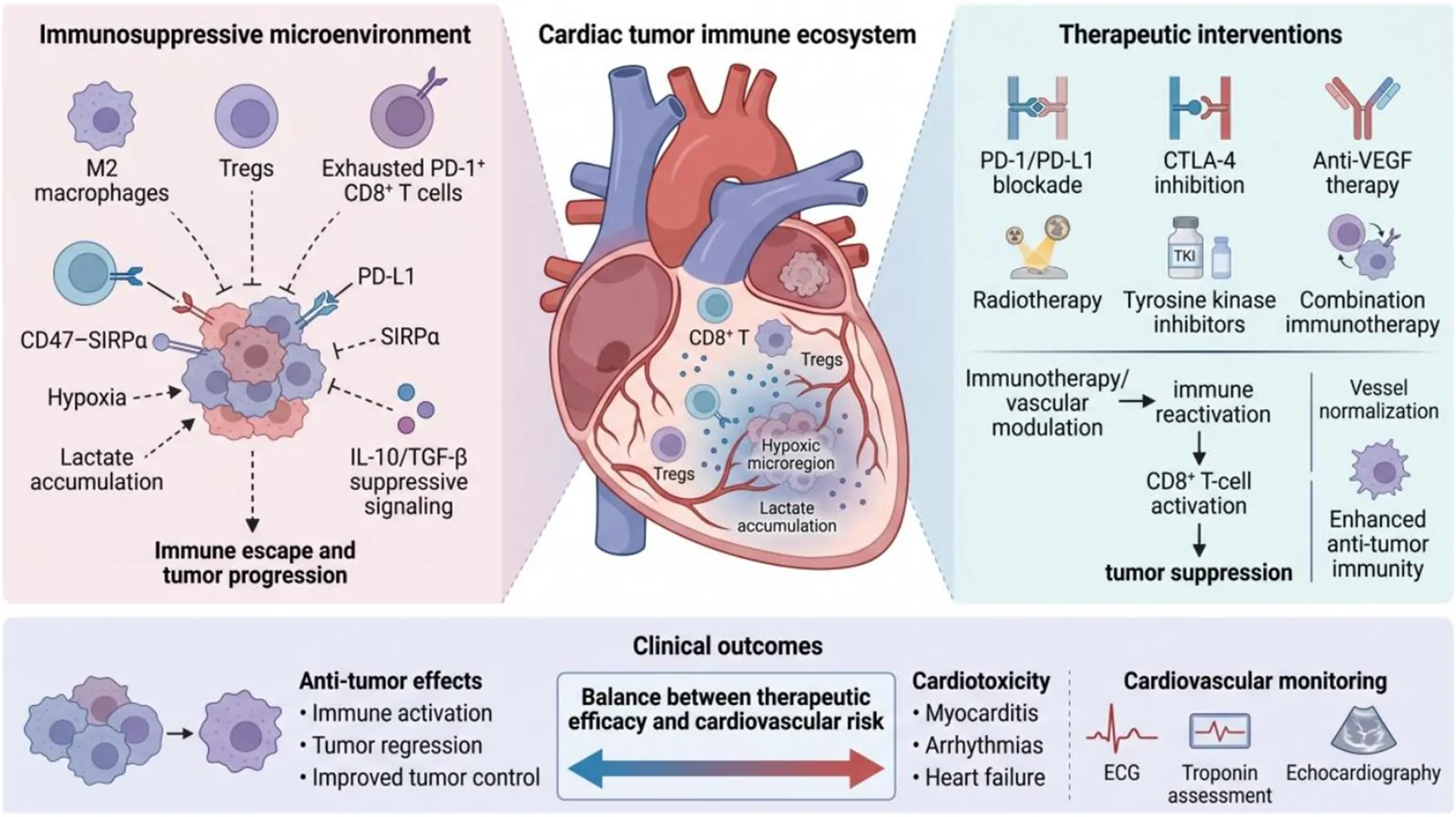
Cardio-immuno-oncology framework in cardiac tumors and cardiac metastases. The cardiac tumor immune ecosystem is shaped by interactions among tumor cells, immune cells, cytokine gradients, hypoxic regions, metabolic stress, and the vascular microenvironment. Immunosuppressive mechanisms, including M2 macrophages, regulatory T cells, exhausted PD-1+ CD8+ T cells, PD-L1-mediated immune inhibition, CD47–SIRPα signaling, hypoxia, lactate accumulation, and IL-10/TGF-β suppressive signaling, may promote immune escape and tumor progression. Therapeutic interventions, including PD-1/PD-L1 blockade, CTLA-4 inhibition, anti-VEGF therapy, radiotherapy, tyrosine kinase inhibitors (TKIs), and combination immunotherapy, may restore antitumor immune activity, enhance CD8+ T-cell function, normalize tumor vasculature, and promote tumor suppression. However, therapeutic immune activation must be balanced against cardiovascular risk, including myocarditis, arrhythmias, and heart failure, highlighting the need for cardiovascular surveillance with ECG, cardiac troponin assessment, and echocardiography. Several mechanisms depicted in this conceptual framework are supported primarily by evidence from extracardiac tumors or selected cardiac tumor subtypes. In particular, cardiac tumor-specific evidence for CD47–SIRPα signaling is currently limited to cardiac myxoma, whereas direct evidence for lactate-driven immune suppression in cardiac tumors remains limited.

### 3.1 Immune landscape of cardiac myxoma

Cardiac myxoma, the most common primary cardiac tumor, exhibits a distinct immune microenvironment characterized by prominent inflammatory features and extensive stromal–immune interactions. Clinically, patients with cardiac myxoma frequently present with systemic inflammatory manifestations, including fever, elevated erythrocyte sedimentation rate, and constitutional symptoms, suggesting the presence of an active inflammatory milieu (13, 24).

At the tissue level, cardiac myxoma is embedded within a cytokine-rich microenvironment composed of tumor cells, stromal elements, and infiltrating immune cells. This microenvironment may facilitate immune dysregulation and has been proposed to contribute to local lymphoproliferative features. In particular, cardiac myxoma has been proposed to form a relatively confined niche that allows the accumulation of cytokines and immune cells, potentially contributing to impaired immune surveillance (14, 26).

Beyond inflammatory signaling, intracellular processes such as autophagy have also been implicated in shaping the tumor microenvironment of cardiac myxoma. Activation of autophagy-related pathways in both tumor and stromal compartments is associated with cellular stress adaptation, tumor growth, and immune modulation (15, 26). These findings suggest that the myxoma microenvironment is not only immune-active but also metabolically regulated.

Taken together, the immune landscape of cardiac myxoma can be broadly characterized as inflammation-associated and relatively immune-active, with strong involvement of cytokine signaling and innate immune components. Nevertheless, the functional consequences of these immune features remain incompletely understood, and further mechanistic studies are required to clarify their role in tumor progression and immune regulation. Importantly, whether specific immune, inflammatory, or metabolic features are associated with clinically relevant outcomes such as myxoma recurrence or embolic events remains insufficiently established.

### 3.2 Immune landscape of cardiac sarcoma

In contrast to cardiac myxoma, cardiac sarcomas exhibit a markedly different immune profile, generally characterized by immune suppression and limited anti-tumor immune activity, as suggested by single-cell evidence from primary cardiac angiosarcoma and broader sarcoma immune landscape studies (9, 27).

The tumor microenvironment in sarcomas is typically dominated by immunosuppressive mechanisms. Tumor-associated macrophages (TAMs), particularly those with an M2-like phenotype, contribute to tumor progression by promoting tissue remodeling, angiogenesis, and suppression of adaptive immune responses. More broadly, innate and adaptive immune cells engage in bidirectional crosstalk with tumor cells, leading to the establishment of a microenvironment that favors tumor growth and immune evasion (28, 29).

Large-scale transcriptomic analyses have further revealed that sarcomas can be stratified into distinct immune subtypes based on the composition of the tumor microenvironment. These subtypes range from “immune-desert” tumors with minimal immune infiltration to “immune-rich” tumors characterized by the presence of tertiary lymphoid structures (TLS) and B-cell–associated signatures, which are associated with improved survival and enhanced responsiveness to immune checkpoint blockade (30).

Despite this heterogeneity, a substantial proportion of sarcomas exhibit a relatively “cold” immune phenotype, with low levels of effective cytotoxic T-cell infiltration. This limited immunogenicity is thought to contribute to the modest efficacy of immune checkpoint inhibitors observed in clinical settings (17, 31). Overall, the immune landscape of cardiac sarcoma can be described as heterogeneous but predominantly immunosuppressive, with important implications for therapeutic responsiveness.

### 3.3 Mechanisms of immune evasion

The distinct immune landscapes observed in cardiac myxoma and cardiac sarcoma are shaped by multiple interconnected mechanisms that collectively regulate immune activation, immune suppression, and tumor progression. These mechanisms include inhibitory checkpoint pathways, tumor-associated macrophages, metabolic constraints, and cytokine-mediated regulation. Rather than acting independently, these processes form an integrated immune-regulatory network that may enable tumor cells to evade immune surveillance and influence therapeutic response (12, 32, 33). However, direct mechanistic evidence in cardiac tumors remains limited. Therefore, in the following sections, we distinguish observations demonstrated in cardiac tumors from immune-evasion mechanisms that are primarily established in extracardiac malignancies and remain to be validated in cardiac tumor-specific models.

#### 3.3.1 Immune checkpoint pathways

Immune checkpoint pathways suppress anti-tumor T-cell activity by attenuating T-cell receptor signaling and effector function. Among the best-characterized pathways, CTLA-4 primarily limits T-cell priming and activation, whereas PD-1 signaling restrains activated T cells within peripheral tissues and the tumor microenvironment. Tumor or stromal PD-L1 expression can therefore reduce T-cell proliferation, cytokine production, and cytotoxicity and may contribute to dysfunctional or exhausted T-cell states. These mechanisms are well established across multiple solid tumors, although their relative importance and regulation in individual cardiac tumor types remain incompletely defined (32–34).

In cardiac tumors, the extent to which individual checkpoint pathways dominate immune escape remains tumor-type dependent and incompletely characterized. In cardiac myxoma specifically, recent single-cell and proteomic evidence has implicated the SIRPα–CD47 axis as a potential phagocytic immune checkpoint (12). Interaction between tumor-associated CD47 and SIRPα on myeloid cells can transmit an inhibitory “do-not-eat-me” signal that limits phagocytic clearance. Importantly, current evidence for this pathway in the cardiac tumor setting is derived from cardiac myxoma, and its relevance to primary cardiac sarcomas or cardiac metastases has not yet been established.

#### 3.3.2 Tumor-associated macrophages

Tumor-associated macrophages (TAMs) are major regulators of the immune microenvironment in many solid tumors and can promote angiogenesis, extracellular-matrix remodeling, tumor-cell invasion, and suppression of adaptive immunity. Their abundance and functional state are highly context dependent, and direct evidence in cardiac tumors remains substantially more limited than in common extracardiac malignancies. Rather than mediating anti-tumor immunity, TAMs are frequently polarized toward phenotypes that support tumor progression.

TAMs contribute to immune evasion through multiple mechanisms. They promote tumor growth and metastasis by enhancing angiogenesis, facilitating tumor cell invasion, and remodeling the extracellular matrix. At the same time, TAMs suppress adaptive immune responses by inhibiting T-cell activation and promoting immunoregulatory signaling pathways (29). Cardiac tumor-specific evidence is beginning to support this concept. Single-cell analysis of primary cardiac angiosarcoma identified suppressive macrophage populations, including SPP1+ and OLR1+ macrophages (9). These findings suggest that myeloid-cell-rich immune niches may contribute to local immune suppression in cardiac angiosarcoma and provide a more direct mechanistic basis for considering macrophage-mediated immune regulation in this tumor type. However, whether these macrophage states are conserved across other cardiac sarcoma subtypes or cardiac metastases remains unknown.

Accordingly, TAM-mediated immune suppression appears biologically plausible in cardiac tumors, but the current evidence is strongest for selected cardiac sarcoma datasets rather than for cardiac tumors as a homogeneous group. Important unresolved questions include whether specific macrophage states correlate with immune checkpoint expression, treatment response, angiogenic activity, or clinical outcome, and whether these populations represent therapeutically actionable targets.

#### 3.3.3 Metabolic reprogramming

Metabolic reprogramming constitutes an important and increasingly recognized mechanism of tumor immune evasion. Tumor cells undergo extensive metabolic alterations that enable them to sustain rapid proliferation while simultaneously reshaping the microenvironment to impair immune cell function.

A hallmark of tumor metabolism is the increased uptake and utilization of nutrients such as glucose and amino acids, even under conditions of limited availability. These changes not only support tumor growth but also influence the composition and function of the tumor microenvironment (35).

Critically, tumor cells and immune cells compete for metabolic resources within the tumor microenvironment. The high metabolic demand of proliferating tumor cells can deplete essential nutrients, thereby limiting T-cell activation and effector function. In addition, metabolic byproducts such as lactate accumulate within the tumor microenvironment and directly suppress immune cell activity, impairing cytokine production and cytotoxic responses (36).

These metabolic mechanisms are well established in many extracardiac solid tumors, where glucose competition, lactate accumulation, hypoxia, and other metabolic constraints can impair T-cell and myeloid-cell function (35, 36). Their relevance to cardiac tumors, however, remains much less certain. In cardiac myxoma, glycolysis-associated tumor biology and immune-evasion features have been reported (12), providing preliminary evidence that metabolic regulation may contribute to the local immune microenvironment. Nevertheless, direct *in vivo* evidence demonstrating that glucose depletion or lactate accumulation drives immune suppression in cardiac myxoma, cardiac sarcoma, or cardiac metastases remains limited. These mechanisms should therefore be regarded as biologically plausible and hypothesis-generating rather than established cardiac tumor-specific pathways.

#### 3.3.4 Cytokine signaling

Cytokine signaling provides a major route of communication among tumor cells, stromal cells, and infiltrating immune populations. Depending on cellular source, receptor expression, concentration, and temporal context, cytokines can either support anti-tumor immunity or promote chronic inflammation, immune suppression, angiogenesis, and tumor progression (37, 38). Persistent cytokine signaling can therefore reshape both tumor-cell behavior and the composition and functional state of the immune microenvironment.

Cardiac myxoma provides one of the clearer cardiac tumor-specific examples of cytokine-mediated regulation. Myxoma cells can produce IL-6, and IL-6-associated activation of STAT3 and Akt signaling may further reinforce inflammatory signaling within the tumor microenvironment (14). This mechanism may help explain the prominent inflammatory phenotype associated with some cardiac myxomas. However, direct evidence linking this signaling program to immune-cell dysfunction, recurrence, embolic events, or treatment response remains limited.

## 4 Immunotherapy in cardiac tumors and cardiac metastases

Immunotherapy has emerged as a promising therapeutic strategy in oncology, but its application in cardiac tumors remains limited due to the rarity of these malignancies and the scarcity of dedicated clinical studies. Current evidence suggests that while immune checkpoint inhibitors (ICIs) may provide clinical benefit in selected patients, overall efficacy is modest and highly heterogeneous. These outcomes are closely linked to tumor-intrinsic factors and the immune microenvironment, including immune cell infiltration, vascular abnormalities, and immune regulatory pathways.

### 4.1 Immune checkpoint inhibitors in cardiac sarcoma

Clinical evidence for immunotherapy in primary malignant cardiac tumors is mainly derived from studies of primary cardiac soft tissue sarcomas (1, 6). A recent multicenter retrospective study evaluating ICIs in patients with primary cardiac soft tissue sarcomas demonstrated limited overall efficacy, with an objective response rate of 11.1%, a median progression-free survival of 5.7 months, and a median overall survival of 14.9 months (17). Although durable responses were observed in a subset of patients, these findings highlight the overall modest activity of ICIs in this setting.

Similar trends have been reported in broader soft tissue sarcoma cohorts. In the SARC028 phase II trial, pembrolizumab demonstrated variable clinical activity across sarcoma subtypes, with responses mainly observed in selected soft tissue sarcoma subtypes rather than uniformly across all sarcomas (31). These findings suggest that intrinsic tumor characteristics and immune contexture play critical roles in determining therapeutic responsiveness.

Collectively, current data indicate that ICIs can induce meaningful responses in a subset of patients with primary cardiac sarcomas, but overall activity remains modest and heterogeneous. Interpretation is limited by the retrospective design, small sample size, histological heterogeneity, and absence of prospectively validated cardiac sarcoma-specific predictive biomarkers. The observed limited sensitivity may reflect, at least in part, immunosuppressive tumor microenvironments, restricted effective T-cell infiltration, and tumor-intrinsic heterogeneity; however, these mechanisms have not been prospectively established as determinants of ICI resistance specifically in cardiac sarcoma.

### 4.2 Immunotherapy for cardiac metastasis

With the expanding use of immune checkpoint inhibitors, an increasing number of patients with metastatic cancers and cardiac involvement may receive immunotherapy. Current evidence suggests that ICIs can be administered to selected patients with cardiac metastases, although careful cardiovascular assessment is required. A multicenter international retrospective study of patients with cardiac metastases treated with ICIs showed that immunotherapy could achieve responses in both cardiac lesions and extracardiac tumor burden, particularly in melanoma and non-small cell lung cancer. Importantly, this study did not demonstrate a clear increase in myocarditis or pericarditis rates among patients with cardiac metastases receiving ICIs, suggesting that cardiac involvement alone should not necessarily preclude immunotherapy (18). However, these findings should not be interpreted as evidence of equivalent cardiovascular risk between patients with and without cardiac metastases. The retrospective design is subject to selection bias, limited sample size, heterogeneity in tumor type and cardiac involvement, and the absence of centralized imaging and pathological adjudication. Accordingly, the available data primarily support the view that cardiac metastasis should not be considered an absolute contraindication to ICI therapy, rather than establishing cardiovascular safety equivalence.

Evidence from specific tumor types further supports the potential role of immunotherapy in selected cases of cardiac metastasis. In a case-control study and literature review of metastatic Merkel cell carcinoma with cardiac involvement, cardiac lesions frequently responded to immunotherapy and/or cardiac radiotherapy, and cardiac metastasis was not associated with worse survival compared with non-cardiac distant metastasis (19). Similarly, a case report of myocardial metastasis from nasopharyngeal carcinoma showed a sustained response to anti-PD-1 therapy in a tumor expressing PD-L1 (25). Although these findings are limited by retrospective design and case-level evidence, they suggest that immunotherapy may be a feasible therapeutic option for selected patients with cardiac metastases, especially when the primary tumor is known to be immunotherapy-sensitive.

### 4.3 Combination strategies

Given the limited efficacy of ICI monotherapy, combination strategies have been proposed to enhance therapeutic responses by targeting multiple components of the tumor microenvironment. One important approach involves combining ICIs with antiangiogenic therapies.

Tumor vasculature plays a critical role in regulating immune cell infiltration and function. Abnormal tumor blood vessels can restrict immune cell access and contribute to an immunosuppressive microenvironment. Antiangiogenic agents can normalize tumor vasculature, thereby improving perfusion and facilitating the infiltration of immune effector cells into tumor tissues (39). This vascular normalization can convert an immunosuppressive microenvironment into a more immunologically active state, enhancing the effectiveness of immunotherapy.

In addition to vascular-targeted approaches, combination strategies may include chemotherapy or other targeted therapies that promote antigen release and immune activation. These approaches aim to overcome primary resistance by transforming immunologically “cold” tumors into “hot” tumors that are more responsive to immune checkpoint blockade. Although evidence remains limited, a case report of primary cardiac angiosarcoma described clinical benefit from radiotherapy combined with anti-PD-1 therapy and tyrosine kinase inhibition in a tumor characterized by PD-L1 expression and TLS (16). In cardiac metastases, systemic immunotherapy may also be combined with local radiotherapy in selected cases, but treatment should be individualized because cardiac irradiation may increase the risk of radiation-related cardiac injury, especially when lesions are close to critical cardiac structures (19, 22).

### 4.4 Biomarkers of response

Predictive biomarker development is an important goal for immunotherapy in cardiac tumors; however, no biomarker has yet been prospectively validated specifically for treatment selection in primary cardiac tumors or cardiac metastases. Most candidate markers are derived from broader solid-tumor or sarcoma studies and should therefore be interpreted as potentially informative rather than clinically established in the cardiac tumor setting.

The PD-1/PD-L1 axis represents a central mechanism of immune evasion, and its expression has been widely investigated as a predictive biomarker for immunotherapy response. This pathway functions by inhibiting T-cell activation and promoting immune tolerance within the tumor microenvironment (40). However, PD-L1 expression alone is insufficient to fully predict clinical outcomes, highlighting the need for additional markers.

Tumor-infiltrating lymphocytes (TILs), particularly CD8+ T cells, are strongly associated with response to immune checkpoint blockade. Pre-existing CD8+ T cells within the tumor microenvironment have been shown to predict therapeutic efficacy, as these cells can be reactivated upon checkpoint inhibition (34).

More recently, TLS and B-cell–rich immune niches have emerged as important determinants of response. In soft tissue sarcoma, tumors enriched in B cells and TLS exhibit improved survival and higher response rates to PD-1 blockade (30). These findings suggest that coordinated immune responses involving both T cells and B cells are critical for effective anti-tumor immunity.

Taken together, PD-L1 expression, T-cell infiltration, TLS, and B-cell-rich immune niches represent candidate biomarkers for future cardiac tumor studies rather than validated tools for routine treatment selection. Prospective multicenter validation will be required before biomarker-guided immunotherapy can be incorporated into clinical management of cardiac tumors.

## 5 Multi-omics decoding of tumor immunity in cardiac tumors and cardiac metastases

Multi-omics technologies are increasingly important for decoding tumor immunity in cardiac tumors and cardiac metastases. Because primary cardiac tumors are rare and cardiac tumor tissues are often difficult to obtain, conventional histopathology and bulk molecular profiling may not fully capture the cellular diversity, spatial immune organization, and metabolic features of the tumor microenvironment. Single-cell sequencing, spatial transcriptomics, proteomics, metabolomics, and integrative multi-omics approaches provide complementary layers of information that can reveal tumor–immune interactions, immune-evasion pathways, and potential therapeutic vulnerabilities. The multi-omics framework for decoding tumor immunity and supporting precision cardio-immuno-oncology is summarized in **Figure 3**.

**Figure 3:**
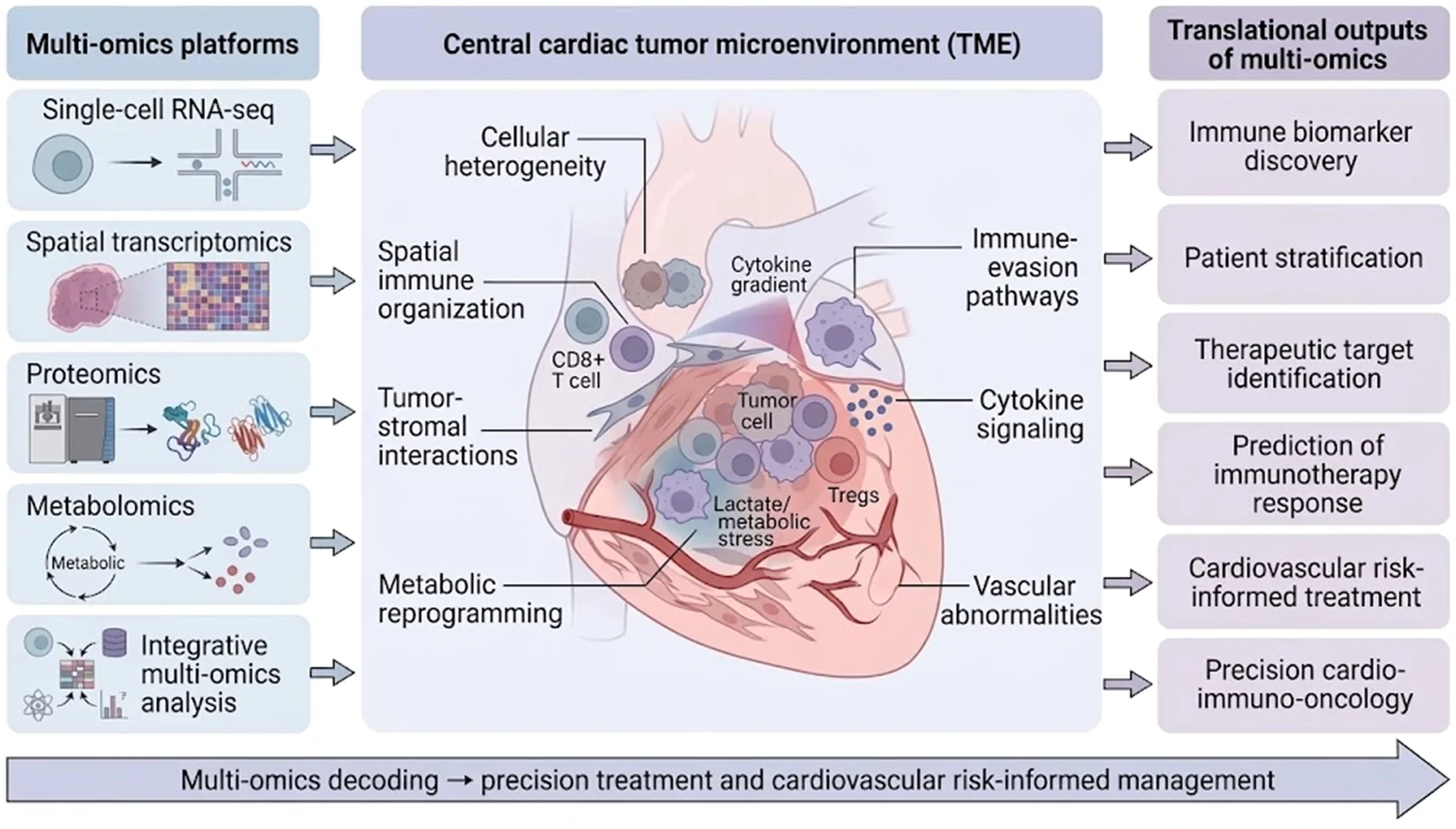
Multi-omics decoding of tumor immunity in cardiac tumors and cardiac metastases. Multi-omics approaches, including single-cell RNA sequencing, spatial transcriptomics, proteomics, metabolomics, and integrative multi-omics analysis, provide complementary insights into the cardiac tumor microenvironment. These technologies help reveal cellular heterogeneity, spatial immune organization, tumor–stromal interactions, immune-evasion pathways, cytokine signaling, metabolic reprogramming, and vascular abnormalities. Integration of these findings may facilitate immune biomarker discovery, patient stratification, therapeutic target identification, prediction of immunotherapy response, and cardiovascular risk-informed treatment, thereby supporting precision cardio-immuno-oncology strategies in cardiac tumors and cardiac metastases.

### 5.1 Single-cell and spatial transcriptomic profiling

Recent advances in single-cell and spatially resolved technologies have substantially improved the characterization of tumor immune microenvironments by enabling high-resolution analysis of cellular composition and organization. Conventional bulk approaches provide limited insight into cellular heterogeneity, whereas emerging techniques allow for precise delineation of immune–tumor interactions within the TME (8).

Importantly, immune cells are not randomly distributed within tumors but instead occupy distinct anatomical and functional regions, such as the tumor core, invasive margin, and TLS. These spatial patterns critically influence immune cell function and their capacity to exert anti-tumor effects (7). For example, cytotoxic T cells localized within the tumor core are more likely to mediate effective anti-tumor responses, whereas exclusion from tumor parenchyma is associated with impaired immune activity.

In addition, tumor microenvironments can be broadly categorized into immune-inflamed, immune-excluded, and immune-desert phenotypes based on both cellular composition and spatial organization. These phenotypes are closely associated with therapeutic outcomes, particularly in the context of immune checkpoint blockade (8).

In cardiac tumors, single-cell and spatial transcriptomic studies have begun to reveal tumor-specific immune architectures. In primary cardiac angiosarcoma, single-cell RNA sequencing has identified intratumoral heterogeneity, suppressive macrophage populations, and immune-suppressive niches (9). In cardiac myxoma, single-cell and spatial transcriptomic analyses have revealed heterogeneous tumor microenvironments, tumor–stromal interactions, and spatially organized immune features (10). Integrated single-cell and proteomic analysis has further suggested the involvement of SIRPα–CD47 signaling and glycolysis-associated immune-evasion features in cardiac myxoma (12). These findings suggest that spatially resolved and multi-layered immune profiling can help explain why different cardiac tumors exhibit distinct immune behavior and therapeutic vulnerability.

### 5.2 Proteomic and metabolomic insights

Proteomic and metabolomic approaches provide additional layers of information beyond transcriptomic profiling. Proteomics can identify functional immune checkpoints, signaling proteins, and tumor–immune interaction pathways, whereas metabolomics can reveal metabolic programs that shape immune cell function within the tumor microenvironment. In cardiac myxoma, integrated single-cell RNA sequencing and proteomic analysis implicated the SIRPα–CD47 immune checkpoint axis and glycolysis-associated immune-evasion features, suggesting that immune escape and metabolic remodeling may be linked in this traditionally benign tumor (12). In addition, multi-omics analysis has provided new information regarding the origin and molecular features of cardiac myxoma, supporting the value of integrative profiling in understanding cardiac tumor biology (11).

These findings are important because they suggest that cardiac tumors should not be viewed only through histological classification. Instead, multi-layered molecular profiling may help identify immune-regulatory pathways, metabolic vulnerabilities, and potential therapeutic targets that are not apparent from conventional pathology alone.

### 5.3 Multi-omics-derived immune biomarkers

Multi-omics profiling may also support the discovery of immune biomarkers for treatment stratification. Potential biomarkers include immune checkpoint expression, CD8+ T-cell infiltration, tumor-associated macrophage states, B-cell-rich TLS, SIRPα–CD47 signaling, glycolysis-related immune suppression, and cytokine signaling pathways such as IL-6/STAT3/Akt. In sarcoma, B-cell-rich TLS have been associated with improved survival and response to PD-1 blockade, suggesting that spatial immune organization may provide information beyond PD-L1 expression alone (30). In cardiac myxoma, SIRPα–CD47 signaling and glycolysis-related immune evasion may represent candidate mechanisms for future investigation rather than established therapeutic biomarkers (12).

Because cardiac tumors are rare, these candidate biomarkers require validation in larger cohorts and collaborative datasets. Nevertheless, multi-omics-derived biomarkers may provide a foundation for future precision cardio-immuno-oncology by linking immune biology, treatment selection, and cardiovascular risk.

### 5.4 Clinical implications for precision treatment

The clinical value of multi-omics lies in its potential to connect tumor immune biology with therapeutic decision-making. For malignant cardiac tumors, multi-omics profiling may eventually help identify immune-inflamed tumors that are more likely to respond to ICIs, immune-desert tumors that may require combination strategies, and macrophage-rich tumors that may benefit from myeloid-targeted approaches. For cardiac metastases, multi-omics biomarkers from the primary tumor and metastatic lesion may help guide immunotherapy selection, particularly when cardiac involvement complicates risk assessment.

At the same time, precision treatment in cardiac tumors must integrate antitumor efficacy with cardiovascular safety. Multi-omics information should therefore be interpreted together with imaging findings, cardiac function, baseline cardiovascular risk, and the likelihood of treatment-related cardiotoxicity. This integrated approach may help transform cardiac tumor management from empirical therapy toward individualized cardio-immuno-oncology.

Despite this potential, substantial barriers currently limit the clinical translation of multi-omics approaches in cardiac tumors. Tissue acquisition is intrinsically difficult because of disease rarity, anatomical constraints, and the limited availability of fresh or adequately preserved specimens. Most available studies involve small discovery cohorts, and independent validation cohorts are uncommon. In addition, multi-omics analyses remain costly and technically demanding, and differences in sample processing, sequencing platforms, batch effects, spatial resolution, and bioinformatic pipelines can limit reproducibility across centers. At present, these approaches should therefore be viewed primarily as research and biomarker-discovery tools rather than established components of routine clinical decision-making. Multicenter sample sharing, standardized analytical workflows, prospective validation, and clinically feasible turnaround times will be necessary before multi-omics-guided treatment can be implemented in precision cardio-immuno-oncology.

## 6 Cardiotoxicity and cardio-oncology management

Although immunotherapy has expanded therapeutic options for multiple malignancies, immune checkpoint inhibitors (ICIs) can also induce immune-related adverse events involving the cardiovascular system, including myocarditis, pericardial disease, arrhythmias, vasculitis, and non-inflammatory myocardial dysfunction (21–23). Among these toxicities, ICI-associated myocarditis is the most clinically important because it is uncommon but potentially fatal. In the context of cardiac tumors or cardiac metastases, recognition of immunotherapy-related cardiotoxicity is particularly important, as tumor-related cardiac involvement and treatment-related myocardial injury may present with overlapping clinical features.

### 6.1 ICI-associated myocarditis

ICI-associated myocarditis is a rare but severe complication of immune checkpoint blockade. It often occurs early after treatment initiation and may present with nonspecific symptoms such as fatigue, dyspnea, chest discomfort, palpitations, or syncope. Clinical manifestations can include elevated cardiac troponin, electrocardiographic abnormalities, arrhythmias, conduction block, ventricular dysfunction, cardiogenic shock, or sudden cardiac death. Importantly, myocarditis may occur even in patients with preserved left ventricular ejection fraction, and it can coexist with immune-related myositis or myasthenia gravis-like syndromes (20, 23).

A multicenter registry study showed that ICI-associated myocarditis had a median onset of approximately one month after ICI initiation and was associated with a high rate of major adverse cardiac events, including cardiovascular death, cardiogenic shock, cardiac arrest, and complete heart block (20). Pharmacovigilance data further suggest that myocarditis, pericardial disease, and vasculitis can occur as immune-related cardiovascular adverse events during ICI therapy, with myocarditis showing particularly high fatality (23). Therefore, even though the absolute incidence is low, early recognition and prompt management are essential.

In patients with cardiac tumors or cardiac metastases, diagnosis can be especially challenging. Dyspnea, chest discomfort, troponin elevation, arrhythmia, pericardial effusion, or ventricular dysfunction may result from tumor infiltration, intracardiac obstruction, pericardial involvement, prior anticancer therapy, or ICI-associated myocarditis. Therefore, clinicians should maintain a high index of suspicion when new cardiovascular symptoms or biomarker abnormalities develop during immunotherapy.

### 6.2 Mechanisms of ICI-related cardiotoxicity

The mechanisms underlying ICI-related cardiotoxicity are closely related to the physiological role of immune checkpoints in maintaining immune tolerance. Under normal conditions, inhibitory pathways such as PD-1/PD-L1 and CTLA-4 limit excessive T-cell activation and protect tissues from immune-mediated injury. ICIs restore anti-tumor immunity by blocking these inhibitory signals, but this immune activation can also disrupt self-tolerance and trigger off-target tissue inflammation (23, 32, 33).

ICI-associated myocarditis is thought to involve aberrant T-cell activation and immune infiltration within the myocardium. One proposed mechanism is that tumor and cardiac tissues may share antigenic targets, leading to cross-reactive T-cell responses against cardiomyocytes. In addition, blockade of PD-1/PD-L1 signaling may remove protective immune restraints in cardiac tissue, allowing activated T cells and macrophages to mediate myocardial inflammation and injury (21–23).

Inflammatory cytokines and immune effector pathways may further amplify tissue damage. Once myocardial inflammation is initiated, activated immune cells can produce cytokines, chemokines, and cytotoxic mediators that aggravate cardiomyocyte injury, impair conduction pathways, and contribute to myocardial dysfunction. This mechanism explains why ICI myocarditis may be accompanied by arrhythmias, conduction abnormalities, skeletal muscle involvement, or systemic immune-related adverse events (21–23).

### 6.3 Integrated mechanisms of therapy-related cardiovascular injury

Beyond ICI-associated myocarditis, cancer treatment-related cardiovascular injury should be understood as a multicellular and systems-level process rather than as isolated cardiomyocyte damage. Inflammatory signaling, oxidative stress, endothelial and microvascular dysfunction, DNA damage responses, mitochondrial injury, fibrotic remodeling, and senescence-associated pathways can interact across cardiomyocytes, endothelial cells, fibroblasts, and infiltrating immune cells. These processes may be particularly relevant when multiple treatment modalities are combined, because immunotherapy, radiotherapy, antiangiogenic therapy, and other systemic treatments can impose distinct but potentially convergent forms of cardiovascular stress (41).

Radiotherapy provides an important example of this intercellular framework. Radiation exposure can induce DNA damage and oxidative stress in cardiac and vascular cells while promoting inflammatory and profibrotic signaling. In addition to direct injury within irradiated cardiac tissue, treatment-related secreted factors and intercellular communication may propagate cardiovascular effects beyond the initially damaged cell population (42). Such mechanisms provide a broader biological context for cardio-immuno-oncology, in which tumor-directed therapy, immune activation, vascular injury, and cardiac remodeling may interact rather than occur as independent processes. Importantly, the relative contribution of these mechanisms in patients with cardiac tumors or cardiac metastases remains poorly defined and requires dedicated mechanistic and prospective investigation (22).

### 6.4 Clinical monitoring and management

For patients with cardiac tumors or cardiac metastases, cardio-oncology management should begin before immunotherapy, because baseline cardiac involvement may overlap with immune-related cardiotoxicity during treatment and requires structured cardiovascular risk assessment. For patients receiving ICIs, especially those with pre-existing cardiovascular disease, prior cardiotoxic therapies, cardiac tumors, or cardiac metastases, baseline evaluation may include clinical history, physical examination, electrocardiography, cardiac biomarkers such as troponin and natriuretic peptides, and echocardiography when clinically indicated. The 2022 ESC cardio-oncology guidelines recommend structured baseline risk assessment and surveillance for patients receiving potentially cardiotoxic cancer therapies, including immunotherapy (22).

During ICI treatment, new cardiac symptoms, electrocardiographic changes, troponin elevation, or new ventricular dysfunction should prompt early evaluation for ICI-associated myocarditis and other cardiovascular complications. Diagnostic assessment may include serial troponin measurement, electrocardiography, echocardiography, cardiac magnetic resonance imaging (CMR), and, in selected cases, endomyocardial biopsy. Importantly, differential diagnosis should also consider acute coronary syndrome, pulmonary embolism, tumor-related cardiac involvement, pericardial disease, sepsis, and progression of underlying cardiovascular disease (22).

When ICI-associated myocarditis is suspected, ICI therapy should generally be withheld, and patients should undergo urgent cardio-oncology evaluation. For moderate-to-severe cases, hospitalization with continuous cardiac monitoring is usually required because of the risk of malignant arrhythmias and conduction block. High-dose corticosteroids are the main initial treatment, and earlier administration of higher-dose steroids has been associated with improved biomarker and clinical outcomes in observational studies (20, 22).

For steroid-refractory or fulminant myocarditis, escalation of immunosuppression may be required. Potential strategies include additional immunosuppressive agents, and emerging evidence has suggested that abatacept-based approaches, sometimes combined with JAK inhibition such as ruxolitinib, may be considered in severe cases under specialist supervision (43). Because severe ICI myocarditis can overlap with myositis and respiratory muscle involvement, careful assessment for concomitant skeletal muscle or respiratory muscle failure is also important (43).

Overall, clinical management should be individualized according to severity, cancer status, cardiac function, and therapeutic alternatives. Decisions about ICI rechallenge after myocarditis require extreme caution and should be made through multidisciplinary discussion involving oncology, cardiology, and cardio-oncology specialists. For patients with cardiac tumors or cardiac metastases, this multidisciplinary approach is especially important because tumor-related cardiac complications and immune-related cardiotoxicity may be difficult to distinguish clinically (22).

In patients with cardiac metastases, immunotherapy should not be considered absolutely contraindicated solely because of cardiac involvement. However, these patients require particularly careful baseline assessment because pericardial effusion, myocardial infiltration, intracardiac masses, conduction abnormalities, or reduced cardiac reserve may already be present before treatment. During therapy, new dyspnea, chest discomfort, troponin elevation, arrhythmia, or ventricular dysfunction should be evaluated for both tumor-related complications and immune-related cardiotoxicity. Therefore, immunotherapy in patients with cardiac tumors or cardiac metastases should be guided by careful patient selection, baseline cardiovascular risk assessment, and close multidisciplinary surveillance (18, 22).

## 7 Discussion and future perspectives

Despite growing interest in the immune microenvironment and immunotherapy of cardiac tumors, this field remains at an early stage. Current evidence suggests that immune regulation may influence the biology and treatment response of both primary cardiac tumors and cardiac metastases. However, several major challenges still limit clinical translation, including disease rarity, insufficient prospective data, incomplete molecular characterization, and the need to balance antitumor efficacy with cardiovascular safety. The major challenges and future priorities for precision cardio-immuno-oncology are summarized in **Figure 4**.

**Figure 4:**
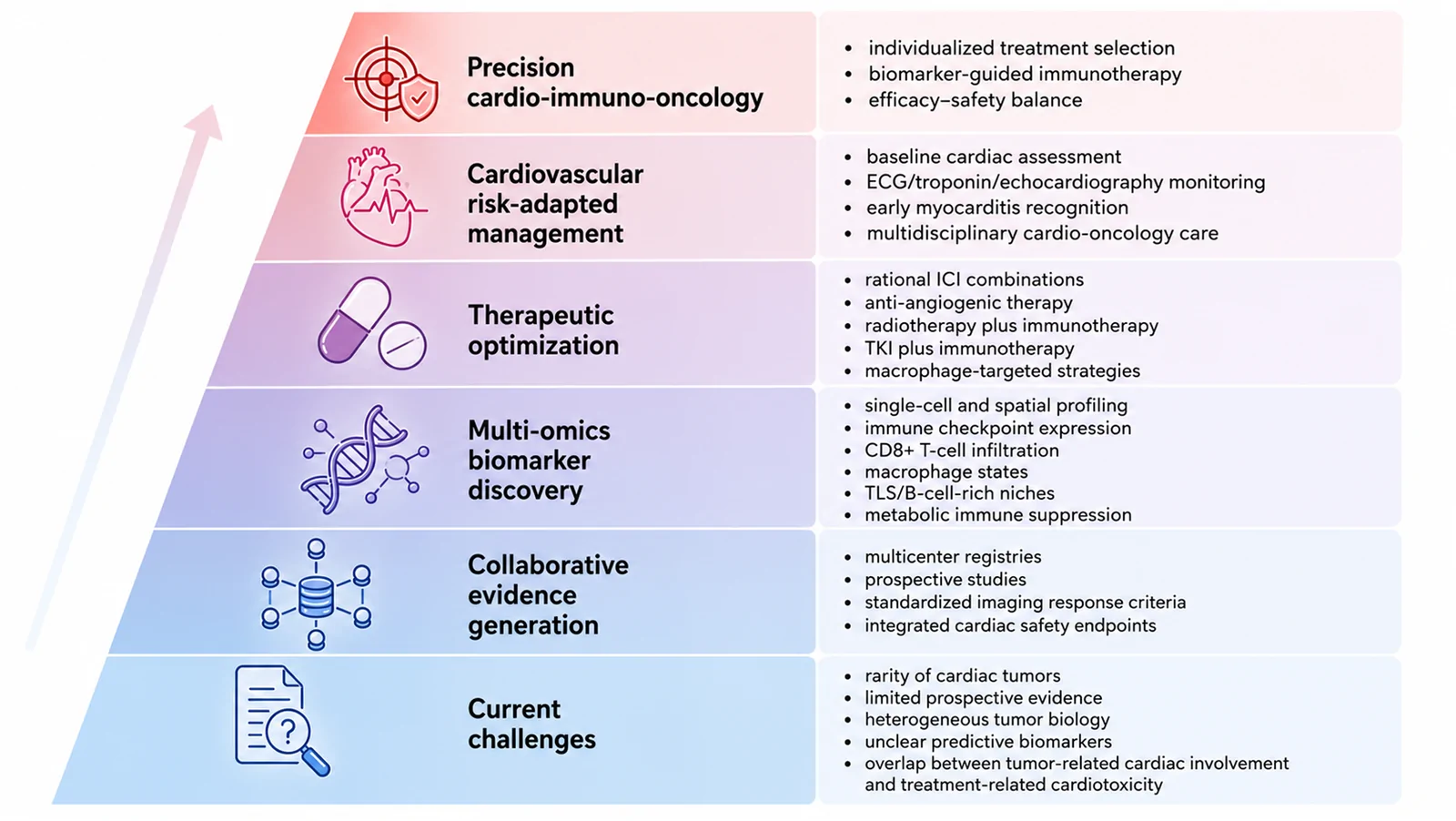
Future roadmap for precision cardio-immuno-oncology in cardiac tumors and cardiac metastases. This roadmap summarizes the major steps required to advance the field from current challenges toward precision cardio-immuno-oncology. Current barriers include the rarity of cardiac tumors, limited prospective evidence, heterogeneous tumor biology, unclear predictive biomarkers, and overlap between tumor-related cardiac involvement and treatment-related cardiotoxicity. Future progress will require collaborative evidence generation, standardized imaging response criteria, integrated cardiac safety endpoints, multi-omics-based biomarker discovery, rational therapeutic optimization, and cardiovascular risk-adapted management. Ultimately, precision cardio-immuno-oncology should integrate individualized treatment selection, biomarker-guided immunotherapy, and careful balancing of antitumor efficacy with cardiovascular safety.

### 7.1 Limitations of the current evidence base

The rarity of cardiac tumors represents the most important obstacle to high-quality clinical research. Primary cardiac tumors are uncommon, and malignant primary cardiac tumors are even rarer. As a result, most available evidence comes from single-center retrospective studies, multicenter retrospective cohorts, small case series, or individual case reports (1, 6). This limitation is particularly evident in primary cardiac sarcomas, where therapeutic decisions are often extrapolated from broader soft tissue sarcoma studies rather than based on dedicated cardiac sarcoma trials (17).

The evidence base for immunotherapy in cardiac tumors is also limited. Although immune checkpoint inhibitors have shown modest activity in primary cardiac soft tissue sarcomas, response rates remain low and heterogeneous (17). Similarly, data on immunotherapy for cardiac metastases are mainly retrospective, although current evidence suggests that selected patients may benefit from ICIs without a clearly observed increase in myocarditis or pericarditis (18). These findings are encouraging but should be interpreted cautiously because of small sample sizes, selection bias, and differences in tumor type, disease stage, prior therapy, and cardiac involvement.

Another challenge is the lack of standardized criteria for evaluating cardiac tumor response. Cardiac motion, tumor necrosis, thrombus formation, pericardial disease, and treatment-related inflammation may complicate imaging interpretation. Therefore, future studies should develop more consistent imaging-based response criteria and integrate cardiovascular outcomes into oncological trials. Collaborative registries and international multicenter studies will be essential to overcome the limitations imposed by disease rarity.

These limitations also constrain the conclusions of the present review. The available literature is heterogeneous in tumor type, study design, treatment exposure, and outcome assessment, and much of the mechanistic interpretation relies on small observational datasets or extrapolation from extracardiac tumors. Publication bias toward unusual or treatment-responsive cases is also possible. Accordingly, several proposed immune mechanisms, biomarkers, and therapeutic strategies discussed in this review should be considered hypothesis-generating until validated in larger and prospectively characterized cardiac tumor cohorts.

### 7.2 Need for collaborative registries and prospective studies

Given the rarity of primary cardiac tumors and the limited availability of well-characterized cohorts of cardiac metastases, collaborative registries and prospective multicenter studies are essential for improving evidence quality. Future studies should collect standardized data on tumor histology, cardiac involvement, imaging characteristics, immune biomarkers, multi-omics profiles, treatment response, cardiovascular adverse events, and survival outcomes. Such datasets would help validate candidate biomarkers, refine response criteria for cardiac lesions, and support safer integration of immunotherapy into the management of patients with cardiac involvement.

### 7.3 Future therapeutic strategies

Current immunotherapy strategies for cardiac tumors remain limited, and future treatment will likely require rational combinations. Immune checkpoint blockade alone has shown only modest efficacy in primary cardiac soft tissue sarcomas (17). Therefore, combination strategies that remodel the tumor microenvironment may be necessary to improve response.

One potential direction is combining ICIs with antiangiogenic therapy. Abnormal tumor vasculature can restrict immune-cell infiltration and promote immune suppression, whereas antiangiogenic agents may normalize tumor vessels and enhance immune effector-cell entry into tumors (39). In primary cardiac angiosarcoma, a case report described clinical benefit from radiotherapy combined with anti-PD-1 therapy and tyrosine kinase inhibition in a tumor characterized by PD-L1 expression and TLS (16). Although this evidence remains preliminary, it supports the rationale for exploring multimodal treatment rather than relying solely on single-agent immunotherapy in selected patients.

Another future direction is targeting non–T-cell components of the immune microenvironment. Tumor-associated macrophages are central mediators of tumor-promoting inflammation, angiogenesis, invasion, and immune suppression, and may represent important therapeutic targets (28, 29). In cardiac tumors, suppressive macrophage populations have been identified in primary cardiac angiosarcoma, while macrophage infiltration and macrophage–tumor interactions have also been implicated in cardiac myxoma biology (9, 13, 44). Therefore, macrophage-targeted strategies may be relevant to both malignant and benign cardiac tumors.

In cardiac myxoma, emerging mechanisms such as SIRPα–CD47 immune checkpoint signaling, glycolysis-associated immune-evasion features, autophagy-related inflammatory regulation, and IL-6/STAT3/Akt signaling may provide future therapeutic directions (12, 14, 15). These targets remain experimental, and surgical resection is still the standard treatment for cardiac myxoma. However, mechanistic studies may be valuable for understanding recurrence, embolic risk, systemic inflammation, and potential non-surgical strategies for patients who are not ideal surgical candidates.

### 7.4 Precision cardio-immuno-oncology

The future management of cardiac tumors should move toward a precision cardio-immuno-oncology model. This approach should integrate tumor type, immune microenvironment, molecular biomarkers, imaging characteristics, treatment sensitivity, and cardiovascular risk into individualized therapeutic decision-making.

From the oncology perspective, future studies should evaluate whether features such as immune checkpoint expression, CD8+ T-cell infiltration, PD-L1 status, TLS, B-cell-rich immune niches, tumor mutational burden, and microsatellite instability can contribute to treatment stratification. At present, none of these biomarkers has been prospectively validated specifically for treatment selection in cardiac tumors (30, 34, 40). In sarcoma, B-cell-rich TLS have been associated with improved survival and response to PD-1 blockade, suggesting that spatial immune organization may be an important biomarker beyond PD-L1 expression alone (30).

From the cardiovascular perspective, immunotherapy should be accompanied by careful cardiac risk assessment and surveillance. This is especially important in patients with primary cardiac tumors or cardiac metastases, because pre-existing cardiac involvement may overlap with ICI-related myocarditis, pericardial disease, arrhythmias, or myocardial dysfunction. Baseline electrocardiography, cardiac biomarkers, echocardiography, and advanced imaging should be considered according to risk level and clinical context (22). Early recognition of immune-related cardiotoxicity is essential because ICI-associated myocarditis may be rare but severe and potentially fatal (20, 23).

Ultimately, precision cardio-immuno-oncology requires close collaboration among oncologists, cardiologists, cardiac surgeons, radiologists, pathologists, and translational researchers. Future clinical studies should not only evaluate antitumor efficacy but also incorporate cardiac safety endpoints, immune biomarkers, imaging response criteria, and patient-centered outcomes. By integrating immune biology with cardiovascular safety, this approach may improve the management of cardiac tumors and cardiac metastases while reducing the risks associated with immunotherapy.

## 8 Summary

Primary cardiac tumors are rare, whereas cardiac metastases are comparatively more common forms of cardiac involvement in patients with advanced malignancies; both are clinically important because of their potential cardiovascular consequences. Although the heart has traditionally been considered an uncommon site for malignant tumor development, tumor involvement of the heart can cause disproportionate clinical harm through obstruction, embolism, arrhythmias, pericardial disease, myocardial infiltration, and overlap with treatment-related cardiotoxicity.

Emerging evidence indicates that cardiac tumors possess distinct immune-regulated features. Cardiac myxoma may exhibit inflammatory, cytokine-mediated, metabolic, and candidate immune-evasion programs, whereas malignant cardiac tumors, particularly primary cardiac sarcomas such as cardiac angiosarcoma, are often associated with immunosuppressive niches, macrophage-rich microenvironments, T-cell dysfunction, and heterogeneous sensitivity to immune checkpoint blockade. Multi-omics approaches, including single-cell sequencing, spatial transcriptomics, proteomics, and metabolomics, provide new opportunities to decode these immune landscapes and identify therapeutic vulnerabilities.

The use of immunotherapy in patients with cardiac tumors or cardiac metastases requires careful cardiovascular consideration, because immune checkpoint inhibitors may cause severe cardiovascular toxicities, particularly myocarditis, and these complications may be difficult to distinguish from tumor-related cardiac involvement. Future progress will depend on collaborative registries, multicenter studies, multi-omics-based biomarker discovery, rational combination therapies, and cardio-oncology surveillance strategies. By integrating tumor immune biology with cardiovascular safety, precision cardio-immuno-oncology may improve patient stratification, therapeutic efficacy, and clinical outcomes.

## References

[1] Tyebally S Chen D Bhattacharyya S Mughrabi A Hussain Z Manisty C . Cardiac tumors: Jacc cardiooncology state-of-the-art review. JACC: CardioOncology. (2020) 2:293–311. doi: 10.1016/j.jaccao.2020.05.009 34396236 PMC8352246

[2] Lin Z Xiao H Liu J Chen L Zhang H Huang Y . Clinical features and prognosis of cardiac metastatic tumors. BMC Cancer. (2023) 23:1235. doi: 10.1186/s12885-023-11733-1 38102550 PMC10722672

[3] Butany J Nair V Naseemuddin A Nair GM Catton C Yau T . Cardiac tumours: Diagnosis and management. Lancet Oncol. (2005) 6:219–28. doi: 10.1016/s1470-2045(05)70093-0 15811617

[4] Leja MJ Shah DJ Reardon MJ . Primary cardiac tumors. Texas Heart Institute J. (2011) 38:261–2. PMC3113129 21720466

[5] Lestuzzi C . Primary tumors of the heart. Curr Opin Cardiol. (2016) 31:593–8. doi: 10.1097/HCO.0000000000000335 27652810

[6] Lefler DS Nassif Haddad EF Nohria A Agulnik M Crane J Fradley MG . Sarcoma: Cardiovascular and oncologic considerations: Jacc: Cardiooncology state-of-the-art review. JACC: CardioOncology. (2025) 7:800–15. doi: 10.1016/j.jaccao.2025.09.005 41247330 PMC12805396

[7] Fridman WH Pages F Sautes-Fridman C Galon J . The immune contexture in human tumours: Impact on clinical outcome. Nat Rev Cancer. (2012) 12:298–306. doi: 10.1038/nrc3245 22419253

[8] Binnewies M Roberts EW Kersten K Chan V Fearon DF Merad M . Understanding the tumor immune microenvironment (Time) for effective therapy. Nat Med. (2018) 24:541–50. doi: 10.1038/s41591-018-0014-x 29686425 PMC5998822

[9] Huo J Wang Z Zhao W Chen M Li H He F . Investigating intra-tumoural heterogeneity and microenvironment diversity in primary cardiac angiosarcoma through single-cell rna sequencing. Clin Transl Med. (2024) 14:e70113. doi: 10.1002/ctm2.70113 39658531 PMC11631565

[10] Liu X Shen H Yu J Luo F Li T Li Q . Resolving the heterogeneous tumour microenvironment in cardiac myxoma through single-cell and spatial transcriptomics. Clin Transl Med. (2024) 14:e1581. doi: 10.1002/ctm2.1581 38318640 PMC10844892

[11] Liu S Zhang W Sun H Zheng C Huang K Fan C . Multi-omics data reveal the origin of cardiac myxoma. Commun Biol. (2025) 8:1373. doi: 10.1038/s42003-025-08752-y 41006804 PMC12474904

[12] Zhu X Gao Q Dai X Sun B Li Y Zhai H . Single-cell rna sequencing and proteomics uncover sirpα-cd47 immune checkpoint and glycolysis-driven immune evasion in cardiac myxoma. Front Immunol. (2025) 16:1652645. doi: 10.3389/fimmu.2025.1652645 40959090 PMC12433983

[13] Di Vito A Santise G Mignogna C Chiefari E Cardillo G Presta I . Innate immunity in cardiac myxomas and its pathological and clinical correlations. Innate Immun. (2018) 24:47–53. doi: 10.1177/1753425917741678 29145790 PMC6830761

[14] Jougasaki M Takenoshita Y Umebashi K Yamamoto M Sudou K Nakashima H . Autocrine regulation of interleukin-6 via the activation of stat3 and akt in cardiac myxoma cells. Int J Mol Sci. (2024) 25:2232. doi: 10.3390/ijms25042232 38396907 PMC10888597

[15] Sramek V Dridi M Papoudou-Bai A Dumollard JM Peoc'h M Karpathiou G . Autophagy in cardiac myxoma: An important puzzle piece in understanding its inflammatory environment. Pathol Res Pract. (2021) 226:153609. doi: 10.1016/j.prp.2021.153609 34537518

[16] Deng S Yang X He L Zhang Q Zhao C Meng H . Radiotherapy combined with anti-pd-1 and tki for primary cardiac angiosarcoma considering the joint assessment of tlss and pd-l1: A case report. J Cardiothoracic Surg. (2024) 19:194. doi: 10.1186/s13019-024-02752-5 38594687 PMC11003096

[17] Nassar AH El-Am E Denu R Abou Alaiwi S El Zarif T Macaron W . Clinical outcomes among immunotherapy-treated patients with primary cardiac soft tissue sarcomas: A multicenter retrospective study. JACC: CardioOncology. (2024) 6:71–9. doi: 10.1016/j.jaccao.2023.11.007 38510282 PMC10950431

[18] Nassar AH Abou Alaiwi S El Zarif T Denu R Macaron W Abdel-Wahab N . Safety and efficacy of immune checkpoint therapy for the treatment of patients with cardiac metastasis: A multicenter international retrospective study. J Immunother Cancer. (2025) 13:e009364. doi: 10.1136/jitc-2024-009364 40032601 PMC11877189

[19] Akaike T Cahill K Akaike G Huynh ET Hippe DS Shinohara MM . Management and prognosis of cardiac metastatic merkel cell carcinoma: A case-control study and literature review. Cancers. (2022) 14:5914. doi: 10.3390/cancers14235914 36497395 PMC9741306

[20] Mahmood SS Fradley MG Cohen JV Nohria A Reynolds KL Heinzerling LM . Myocarditis in patients treated with immune checkpoint inhibitors. J Am Coll Cardiol. (2018) 71:1755–64. doi: 10.1016/j.jacc.2018.02.037 29567210 PMC6196725

[21] Hu J-R Florido R Lipson EJ Naidoo J Ardehali R Tocchetti CG . Cardiovascular toxicities associated with immune checkpoint inhibitors. Cardiovasc Res. (2019) 115:854–68. doi: 10.1093/cvr/cvz026 30715219 PMC6452314

[22] Lyon AR López-Fernández T Couch LS Asteggiano R Aznar MC Bergler-Klein J . 2022 esc guidelines on cardio-oncology developed in collaboration with the european hematology association (Eha), the european society for therapeutic radiology and oncology (Estro) and the international cardio-oncology society (Ic-Os). Eur Heart J. (2022) 43:4229–361. doi: 10.1093/eurheartj/ehac244 36017568

[23] Salem J-E Manouchehri A Moey M Lebrun-Vignes B Bastarache L Pariente A . Cardiovascular toxicities associated with immune checkpoint inhibitors: An observational, retrospective, pharmacovigilance study. Lancet Oncol. (2018) 19:1579–89. doi: 10.1016/s1470-2045(18)30608-9 30442497 PMC6287923

[24] Pinede L Duhaut P Loire R . Clinical presentation of left atrial cardiac myxoma. A series of 112 consecutive cases. Medicine. (2001) 80:159–72. doi: 10.1097/00005792-200105000-00002 11388092

[25] Tang X Zhou W Huang D Chen L Zhang G . Anti-pd-1 therapy-a potential treatment for myocardial metastasis from nasopharyngeal carcinoma: A case report. Front Immunol. (2021) 12:688682. doi: 10.3389/fimmu.2021.688682 34804001 PMC8602087

[26] Jantuan E Chiu B Chiu B Shen F Oudit GY Sergi C . The tumor microenvironment may trigger lymphoproliferation in cardiac myxoma. Transl Oncol. (2021) 14:100911. doi: 10.1016/j.tranon.2020.100911 33129111 PMC7586245

[27] Koumarianou A Duran-Moreno J . The sarcoma immune landscape: Emerging challenges, prognostic significance and prospective impact for immunotherapy approaches. Cancers. (2021) 13:363. doi: 10.3390/cancers13030363 33498238 PMC7863949

[28] Mantovani A Marchesi F Malesci A Laghi L Allavena P . Tumour-associated macrophages as treatment targets in oncology. Nat Rev Clin Oncol. (2017) 14:399–416. doi: 10.1038/nrclinonc.2016.217 28117416 PMC5480600

[29] Qian B-Z Pollard JW . Macrophage diversity enhances tumor progression and metastasis. Cell. (2010) 141:39–51. doi: 10.1016/j.cell.2010.03.014 20371344 PMC4994190

[30] Petitprez F de Reynies A Keung EZ Chen TW Sun CM Calderaro J . B cells are associated with survival and immunotherapy response in sarcoma. Nature. (2020) 577:556–60. doi: 10.1038/s41586-019-1906-8 31942077

[31] Tawbi HA Burgess M Bolejack V Van Tine BA Schuetze SM Hu J . Pembrolizumab in advanced soft-tissue sarcoma and bone sarcoma (Sarc028): A multicentre, two-cohort, single-arm, open-label, phase 2 trial. Lancet Oncol. (2017) 18:1493–501. doi: 10.1016/s1470-2045(17)30624-1 28988646 PMC7939029

[32] Pardoll DM . The blockade of immune checkpoints in cancer immunotherapy. Nat Rev Cancer. (2012) 12:252–64. doi: 10.1038/nrc3239 22437870 PMC4856023

[33] Topalian SL Drake CG Pardoll DM . Immune checkpoint blockade: A common denominator approach to cancer therapy. Cancer Cell. (2015) 27:450–61. doi: 10.1016/j.ccell.2015.03.001 25858804 PMC4400238

[34] Tumeh PC Harview CL Yearley JH Shintaku IP Taylor EJM Robert L . Pd-1 blockade induces responses by inhibiting adaptive immune resistance. Nature. (2014) 515:568–71. doi: 10.1038/nature13954 25428505 PMC4246418

[35] Pavlova NN Thompson CB . The emerging hallmarks of cancer metabolism. Cell Metab. (2016) 23:27–47. doi: 10.1016/j.cmet.2015.12.006 26771115 PMC4715268

[36] Buck MD Sowell RT Kaech SM Pearce EL . Metabolic instruction of immunity. Cell. (2017) 169:570–86. doi: 10.1016/j.cell.2017.04.004 28475890 PMC5648021

[37] Grivennikov SI Greten FR Karin M . Immunity, inflammation, and cancer. Cell. (2010) 140:883–99. doi: 10.1016/j.cell.2010.01.025 20303878 PMC2866629

[38] Dranoff G . Cytokines in cancer pathogenesis and cancer therapy. Nat Rev Cancer. (2004) 4:11–22. doi: 10.1038/nrc1252 14708024

[39] Fukumura D Kloepper J Amoozgar Z Duda DG Jain RK . Enhancing cancer immunotherapy using antiangiogenics: Opportunities and challenges. Nat Rev Clin Oncol. (2018) 15:325–40. doi: 10.1038/nrclinonc.2018.29 29508855 PMC5921900

[40] Topalian SL Taube JM Anders RA Pardoll DM . Mechanism-driven biomarkers to guide immune checkpoint blockade in cancer therapy. Nat Rev Cancer. (2016) 16:275–87. doi: 10.1038/nrc.2016.36 27079802 PMC5381938

[41] Herrmann J . Adverse cardiac effects of cancer therapies: Cardiotoxicity and arrhythmia. Nat Rev Cardiol. (2020) 17:474–502. doi: 10.1038/s41569-020-0348-1 32231332 PMC8782611

[42] Logotheti S Pavlopoulou A Rudsari HK Galow AM Kafali Y Kyrodimos E . Intercellular pathways of cancer treatment-related cardiotoxicity and their therapeutic implications: The paradigm of radiotherapy. Pharmacol Ther. (2024) 260:108670. doi: 10.1016/j.pharmthera.2024.108670 38823489

[43] Salem JE Bretagne M Abbar B Leonard-Louis S Ederhy S Redheuil A . Abatacept/ruxolitinib and screening for concomitant respiratory muscle failure to mitigate fatality of immune-checkpoint inhibitor myocarditis. Cancer Discov. (2023) 13:1100–15. doi: 10.1158/2159-8290.CD-22-1180 36815259

[44] Jiang Z Kang Q Qian H Xu Z Tong H Yang J . Revealing the crucial roles of suppressive immune microenvironment in cardiac myxoma progression. Signal Transduction Targeted Ther. (2024) 9:193. doi: 10.1038/s41392-024-01912-2 39090109 PMC11294589

